# Supplementary material for: Meta-Analysis and Gene Set Enrichment Relative to ER Status Reveal Elevated Activity of MYC and E2F in the “Basal” Breast Cancer Subgroup
Source: PLoS One. 2009 Mar 9;4(3):e4710. doi: 10.1371/journal.pone.0004710 (PMC2650420; doi:10.1371/journal.pone.0004710)
Supplement: Dataset S1 — GSEA results from first screen. The file contains a zipped archive of the HTML results of the second GSEA screen. Reports for enriched or depleted datasets may be accessed from the “index.html” link within the my_analysis.GseaPreranked.1219975950625.rpt.zip ZIP archive. Enrichment plots are available for each of the 50 most enriched and depleted gene sets. (13.32 MB ZIP) [file pone.0004710.s001.zip › my_analysis.GseaPreranked.1219975950625.rpt.zip!//PENG_LEUCINE_DN.html]

Details for gene set PENG\_LEUCINE\_DN[GSEA]

|  || Dataset | zSco.ERneg.to.ERpos\_collapsed |
| Phenotype | NoPhenotypeAvailable |
| Upregulated in class | na\_pos |
| GeneSet | PENG\_LEUCINE\_DN |
| Enrichment Score (ES) | 0.48523158 |
| Normalized Enrichment Score (NES) | 2.1112783 |
| Nominal p-value | 0.0 |
| FDR q-value | 1.1088913E-4 |
| FWER p-Value | 0.0050 |
Table: GSEA Results Summary

  

Fig 1: Enrichment plot: PENG\_LEUCINE\_DN      
 Profile of the Running ES Score & Positions of GeneSet Members on the Rank Ordered List

  

| PROBE | GENE SYMBOL | GENE\_TITLE | RANK IN GENE LIST | RANK METRIC SCORE | RUNNING ES | CORE ENRICHMENT || 1 | CDC20 |  |  | 9 | 7.151 | 0.0220 | Yes |
| 2 | CCNA2 |  |  | 52 | 6.297 | 0.0387 | Yes |
| 3 | LDHB |  |  | 82 | 6.005 | 0.0555 | Yes |
| 4 | TRIP13 |  |  | 96 | 5.890 | 0.0732 | Yes |
| 5 | NOL1 |  |  | 152 | 5.558 | 0.0866 | Yes |
| 6 | PSME4 |  |  | 175 | 5.460 | 0.1022 | Yes |
| 7 | MCM4 |  |  | 180 | 5.436 | 0.1191 | Yes |
| 8 | TFRC |  |  | 259 | 5.071 | 0.1293 | Yes |
| 9 | CTSC |  |  | 300 | 4.932 | 0.1418 | Yes |
| 10 | GART |  |  | 415 | 4.639 | 0.1479 | Yes |
| 11 | SRM |  |  | 435 | 4.575 | 0.1609 | Yes |
| 12 | ME2 |  |  | 452 | 4.503 | 0.1740 | Yes |
| 13 | NOL5A |  |  | 502 | 4.345 | 0.1840 | Yes |
| 14 | TOMM40 |  |  | 561 | 4.216 | 0.1930 | Yes |
| 15 | TUBA1 |  |  | 563 | 4.214 | 0.2062 | Yes |
| 16 | CCT6A |  |  | 566 | 4.210 | 0.2194 | Yes |
| 17 | CYCS |  |  | 624 | 4.086 | 0.2280 | Yes |
| 18 | DDX21 |  |  | 653 | 4.024 | 0.2386 | Yes |
| 19 | AMD1 |  |  | 658 | 4.018 | 0.2510 | Yes |
| 20 | NCL |  |  | 678 | 3.977 | 0.2622 | Yes |
| 21 | MRPS12 |  |  | 751 | 3.842 | 0.2689 | Yes |
| 22 | TCP1 |  |  | 779 | 3.799 | 0.2789 | Yes |
| 23 | ILF3 |  |  | 801 | 3.774 | 0.2892 | Yes |
| 24 | KIAA0179 |  |  | 885 | 3.648 | 0.2945 | Yes |
| 25 | VRK1 |  |  | 939 | 3.570 | 0.3018 | Yes |
| 26 | FDPS |  |  | 949 | 3.555 | 0.3123 | Yes |
| 27 | MRPL3 |  |  | 980 | 3.510 | 0.3212 | Yes |
| 28 | NUTF2 |  |  | 1044 | 3.419 | 0.3272 | Yes |
| 29 | CAD |  |  | 1051 | 3.408 | 0.3376 | Yes |
| 30 | SNRPA1 |  |  | 1064 | 3.399 | 0.3474 | Yes |
| 31 | PSMD14 |  |  | 1069 | 3.392 | 0.3578 | Yes |
| 32 | UNG |  |  | 1070 | 3.390 | 0.3686 | Yes |
| 33 | SSB |  |  | 1124 | 3.331 | 0.3751 | Yes |
| 34 | CCT4 |  |  | 1148 | 3.309 | 0.3838 | Yes |
| 35 | HSPE1 |  |  | 1183 | 3.257 | 0.3916 | Yes |
| 36 | EIF4G1 |  |  | 1224 | 3.214 | 0.3987 | Yes |
| 37 | NP |  |  | 1241 | 3.199 | 0.4076 | Yes |
| 38 | AHSA1 |  |  | 1338 | 3.097 | 0.4101 | Yes |
| 39 | EI24 |  |  | 1564 | 2.887 | 0.4022 | Yes |
| 40 | BYSL |  |  | 1577 | 2.872 | 0.4104 | Yes |
| 41 | CCNB1 |  |  | 1626 | 2.823 | 0.4157 | Yes |
| 42 | LDHA |  |  | 1699 | 2.761 | 0.4190 | Yes |
| 43 | RASSF2 |  |  | 1746 | 2.729 | 0.4241 | Yes |
| 44 | SQLE |  |  | 1821 | 2.677 | 0.4270 | Yes |
| 45 | HPRT1 |  |  | 1947 | 2.573 | 0.4257 | Yes |
| 46 | DYRK1A |  |  | 1954 | 2.568 | 0.4333 | Yes |
| 47 | CSE1L |  |  | 1990 | 2.537 | 0.4387 | Yes |
| 48 | PPIB |  |  | 2018 | 2.514 | 0.4446 | Yes |
| 49 | DNAJB6 |  |  | 2019 | 2.514 | 0.4526 | Yes |
| 50 | VDAC1 |  |  | 2049 | 2.484 | 0.4582 | Yes |
| 51 | EBP |  |  | 2055 | 2.478 | 0.4657 | Yes |
| 52 | LIMK2 |  |  | 2272 | 2.335 | 0.4567 | Yes |
| 53 | EMD |  |  | 2397 | 2.262 | 0.4545 | Yes |
| 54 | CCT5 |  |  | 2398 | 2.262 | 0.4616 | Yes |
| 55 | ACAT2 |  |  | 2472 | 2.215 | 0.4631 | Yes |
| 56 | PSMD1 |  |  | 2710 | 2.086 | 0.4517 | Yes |
| 57 | DDX1 |  |  | 2722 | 2.079 | 0.4575 | Yes |
| 58 | PSMD11 |  |  | 2726 | 2.077 | 0.4638 | Yes |
| 59 | TK1 |  |  | 2730 | 2.076 | 0.4702 | Yes |
| 60 | PSMD2 |  |  | 2759 | 2.063 | 0.4746 | Yes |
| 61 | HSPC111 |  |  | 2760 | 2.062 | 0.4811 | Yes |
| 62 | ADRM1 |  |  | 2792 | 2.043 | 0.4852 | Yes |
| 63 | XPO6 |  |  | 2884 | 1.993 | 0.4846 | No |
| 64 | UMPS |  |  | 3124 | 1.877 | 0.4724 | No |
| 65 | MTHFD1 |  |  | 3258 | 1.814 | 0.4681 | No |
| 66 | PSMB7 |  |  | 3335 | 1.778 | 0.4680 | No |
| 67 | TDG |  |  | 3528 | 1.695 | 0.4588 | No |
| 68 | GGA2 |  |  | 3590 | 1.668 | 0.4594 | No |
| 69 | DHX9 |  |  | 3678 | 1.628 | 0.4580 | No |
| 70 | MMP15 |  |  | 3709 | 1.619 | 0.4608 | No |
| 71 | PSMA5 |  |  | 3924 | 1.544 | 0.4495 | No |
| 72 | POLG |  |  | 3925 | 1.544 | 0.4544 | No |
| 73 | DNAJA1 |  |  | 4099 | 1.474 | 0.4459 | No |
| 74 | RNF126 |  |  | 4255 | 1.411 | 0.4386 | No |
| 75 | PTMS |  |  | 4428 | 1.344 | 0.4298 | No |
| 76 | MRPL12 |  |  | 4601 | 1.286 | 0.4209 | No |
| 77 | EIF4G2 |  |  | 4639 | 1.273 | 0.4221 | No |
| 78 | NUP98 |  |  | 4644 | 1.271 | 0.4258 | No |
| 79 | MLF2 |  |  | 4682 | 1.259 | 0.4270 | No |
| 80 | PSMA7 |  |  | 4890 | 1.191 | 0.4150 | No |
| 81 | PAPOLA |  |  | 4942 | 1.175 | 0.4149 | No |
| 82 | EMP3 |  |  | 4980 | 1.164 | 0.4158 | No |
| 83 | GLO1 |  |  | 4995 | 1.158 | 0.4184 | No |
| 84 | CALR |  |  | 5146 | 1.099 | 0.4105 | No |
| 85 | TCEB1 |  |  | 5401 | 1.010 | 0.3944 | No |
| 86 | TGFB1 |  |  | 5452 | 0.993 | 0.3938 | No |
| 87 | EIF5 |  |  | 5824 | 0.870 | 0.3684 | No |
| 88 | PGK1 |  |  | 5836 | 0.865 | 0.3703 | No |
| 89 | TIMM17A |  |  | 6273 | 0.724 | 0.3395 | No |
| 90 | PSMF1 |  |  | 6332 | 0.708 | 0.3373 | No |
| 91 | IDI1 |  |  | 6517 | 0.645 | 0.3254 | No |
| 92 | SFRS2 |  |  | 6677 | 0.597 | 0.3152 | No |
| 93 | SEC61B |  |  | 6746 | 0.575 | 0.3119 | No |
| 94 | CYC1 |  |  | 6838 | 0.540 | 0.3067 | No |
| 95 | HBD |  |  | 7010 | 0.484 | 0.2953 | No |
| 96 | TNFRSF1A |  |  | 7136 | 0.440 | 0.2872 | No |
| 97 | HSPA5 |  |  | 7169 | 0.427 | 0.2861 | No |
| 98 | PTBP1 |  |  | 7300 | 0.383 | 0.2774 | No |
| 99 | WDR1 |  |  | 7353 | 0.366 | 0.2747 | No |
| 100 | ETF1 |  |  | 7421 | 0.338 | 0.2706 | No |
| 101 | OGFR |  |  | 7668 | 0.241 | 0.2528 | No |
| 102 | NMT1 |  |  | 7789 | 0.193 | 0.2443 | No |
| 103 | BRD2 |  |  | 7827 | 0.179 | 0.2420 | No |
| 104 | PTPN6 |  |  | 8135 | 0.061 | 0.2189 | No |
| 105 | XRCC5 |  |  | 8169 | 0.051 | 0.2166 | No |
| 106 | SF3B4 |  |  | 8173 | 0.049 | 0.2165 | No |
| 107 | CCT2 |  |  | 8514 | -0.085 | 0.1910 | No |
| 108 | TOP1 |  |  | 8568 | -0.109 | 0.1873 | No |
| 109 | HSPA4 |  |  | 8813 | -0.214 | 0.1695 | No |
| 110 | POLRMT |  |  | 8846 | -0.229 | 0.1678 | No |
| 111 | COPB2 |  |  | 8889 | -0.254 | 0.1654 | No |
| 112 | SCD |  |  | 8910 | -0.266 | 0.1647 | No |
| 113 | HSPA8 |  |  | 8998 | -0.311 | 0.1591 | No |
| 114 | ARMET |  |  | 9047 | -0.334 | 0.1565 | No |
| 115 | PFKM |  |  | 9071 | -0.347 | 0.1559 | No |
| 116 | UBE2N |  |  | 9341 | -0.478 | 0.1370 | No |
| 117 | KIAA0152 |  |  | 9353 | -0.485 | 0.1377 | No |
| 118 | EIF2B4 |  |  | 9693 | -0.664 | 0.1141 | No |
| 119 | PSMD3 |  |  | 9828 | -0.743 | 0.1062 | No |
| 120 | GSPT1 |  |  | 9841 | -0.751 | 0.1077 | No |
| 121 | NXF1 |  |  | 9968 | -0.825 | 0.1008 | No |
| 122 | ERH |  |  | 10068 | -0.889 | 0.0961 | No |
| 123 | SLC29A1 |  |  | 10768 | -1.365 | 0.0474 | No |
| 124 | UBC |  |  | 10861 | -1.434 | 0.0449 | No |
| 125 | RBM14 |  |  | 10941 | -1.521 | 0.0437 | No |
| 126 | GCDH |  |  | 11137 | -1.691 | 0.0343 | No |
| 127 | PIK3R3 |  |  | 11340 | -1.868 | 0.0249 | No |
| 128 | FKBP4 |  |  | 11428 | -1.966 | 0.0245 | No |
| 129 | MRPL28 |  |  | 11442 | -1.980 | 0.0298 | No |
| 130 | BST2 |  |  | 12218 | -2.872 | -0.0199 | No |
| 131 | FASN |  |  | 12283 | -2.945 | -0.0154 | No |
| 132 | SPINT2 |  |  | 12305 | -2.969 | -0.0076 | No |
| 133 | ALDOA |  |  | 12439 | -3.164 | -0.0077 | No |
| 134 | ALG8 |  |  | 12656 | -3.534 | -0.0129 | No |
| 135 | FKBP2 |  |  | 12681 | -3.577 | -0.0034 | No |
| 136 | DHRS1 |  |  | 12757 | -3.763 | 0.0028 | No |
| 137 | NBL1 |  |  | 12759 | -3.768 | 0.0147 | No |
| 138 | PDCD4 |  |  | 12873 | -4.072 | 0.0190 | No |
| 139 | DHCR24 |  |  | 13061 | -4.682 | 0.0196 | No |
Table: GSEA details [plain text format]

  

Fig 2: PENG\_LEUCINE\_DN: Random ES distribution      
 Gene set null distribution of ES for **PENG\_LEUCINE\_DN**
